# Bis(3-amino­pyrazine-2-carboxyl­ato-κ^2^ *N* ^1^,*O*)diaqua­manganese(II) mono­hydrate

**DOI:** 10.1107/S1600536810035233

**Published:** 2010-09-08

**Authors:** Shan Gao, Seik Weng Ng

**Affiliations:** a College of Chemistry and Materials Science, Heilongjiang University, Harbin 150080, People’s Republic of China; b Department of Chemistry, University of Malaya, 50603 Kuala Lumpur, Malaysia

## Abstract

In the title compound, [Mn(C_5_H_4_N_3_O_2_)_2_(H_2_O)_2_]·H_2_O, the Mn^II^ cation, located on a twofold rotation axis, is *N*,*O*-chelated by two 3-amino­pyrazine-2-carboxyl­ate anions and coordin­ated by two water mol­ecules in a distorted octa­hedral geometry. The uncoordinated water mol­ecules lies on a twofold rotation axis. Adjacent mol­ecules are linked by O—H⋯O and N—H⋯O hydrogen bonds into a three-dimensional network motif.

## Related literature

For the isostructural magnesium analog, see: Ptasiewicz-Bak & Leciejewicz (1997 ▶); Marsh (2004 ▶).
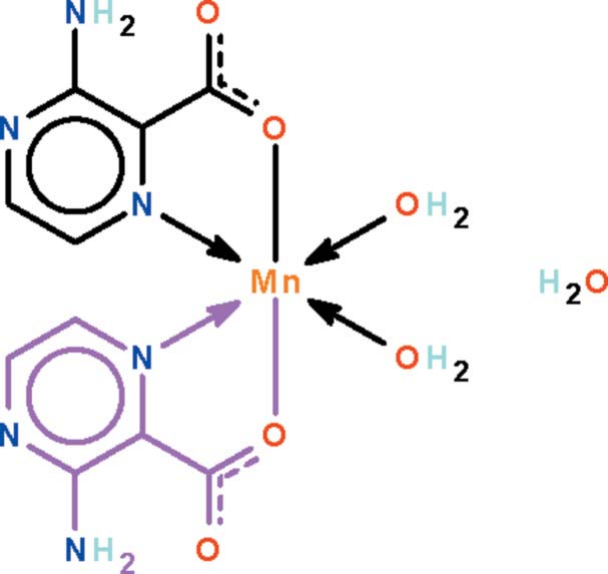


## Experimental

### 

#### Crystal data

[Mn(C_5_H_4_N_3_O_2_)_2_(H_2_O)_2_]·H_2_O
*M* *_r_* = 385.21
Orthorhombic, 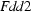

*a* = 8.3107 (6) Å
*b* = 29.5862 (17) Å
*c* = 12.3791 (7) Å
*V* = 3043.8 (3) Å^3^
*Z* = 8
Mo *K*α radiation
μ = 0.92 mm^−1^
*T* = 293 K
0.15 × 0.10 × 0.08 mm

#### Data collection

Rigaku R-AXIS RAPID diffractometer
Absorption correction: multi-scan (*ABSCOR*; Higashi, 1995 ▶) *T*_min_ = 0.875, *T*_max_ = 0.930
7239 measured reflections
1684 independent reflections
1086 reflections with *I* > 2σ(*I*)
*R*_int_ = 0.056

#### Refinement

*R*[*F* ^2^ > 2σ(*F* ^2^)] = 0.047
*wR*(*F* ^2^) = 0.165
*S* = 1.14
1684 reflections
126 parameters
6 restraints
H atoms treated by a mixture of independent and constrained refinement
Δρ_max_ = 0.50 e Å^−3^
Δρ_min_ = −0.90 e Å^−3^
Absolute structure: Flack (1983 ▶), 775 Friedel pairs
Flack parameter: −0.02 (5)

### 

Data collection: *RAPID-AUTO* (Rigaku, 1998 ▶); cell refinement: *RAPID-AUTO*; data reduction: *CrystalStructure* (Rigaku/MSC, 2002 ▶); program(s) used to solve structure: *SHELXS97* (Sheldrick, 2008 ▶); program(s) used to refine structure: *SHELXL97* (Sheldrick, 2008 ▶); molecular graphics: *X-SEED* (Barbour, 2001 ▶); software used to prepare material for publication: *publCIF* (Westrip, 2010 ▶).

## Supplementary Material

Crystal structure: contains datablocks global, I. DOI: 10.1107/S1600536810035233/xu5022sup1.cif

Structure factors: contains datablocks I. DOI: 10.1107/S1600536810035233/xu5022Isup2.hkl

Additional supplementary materials:  crystallographic information; 3D view; checkCIF report

## Figures and Tables

**Table 1:** Hydrogen-bond geometry (Å, °)

*D*—H⋯*A*	*D*—H	H⋯*A*	*D*⋯*A*	*D*—H⋯*A*
O1w—H11⋯O2^i^	0.84 (7)	1.89 (3)	2.704 (7)	162 (9)
O1w—H12⋯N2^ii^	0.84 (7)	2.02 (4)	2.792 (7)	152 (9)
O2w—H2⋯O1	0.84 (7)	2.10 (4)	2.902 (7)	159 (10)
N3—H31⋯O2	0.88 (7)	2.17 (9)	2.690 (8)	118 (8)
N3—H32⋯O2w^iii^	0.88 (3)	2.15 (3)	3.001 (7)	161 (9)

Symmetry codes: (i) 
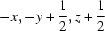
; (ii) 
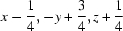
; (iii) 
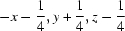
.

**Fig. 1.**
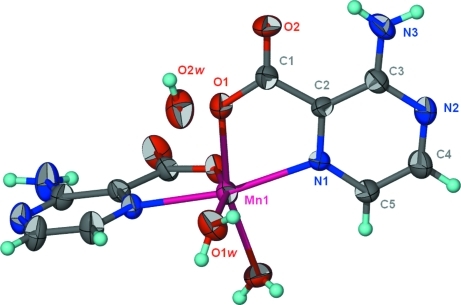
Thermal ellipsoid plot (Barbour, 2001) of Mn(H2O)2(C5H4N3O2)2.H2O at the 50% probability level; hydrogen atoms are drawn as spheres of arbitrary radius. The Mn and lattice water molecule lie on a twofold rotation axis. Symmetry-related atoms are not labeled.

## supplementary crystallographic information

### Comment

The crystal structure of Mg(H_2_O)_2_(C_5_H_4_N_3_O_2_)_2_^.^H_2_O was described in the *Cc* space group (Ptasiewicz-Bak & Leciejewicz, 1997); the space group was revised to the *Fdd*2 space group (Marsh, 2004). The manganese analog (Scheme I) is isostructural; The water-coordinated manganese atom is *N*,*O*-chelated by the carboxylate ion (Fig. 2) in an octahedral environment. The mononuclear and lattice water both lie on a twofold rotation axis. Adjacent molecules are linked by O–H···O and N–H···O hydrogen bonds into a three-dimensional network motif.

### Experimental

Manganese acetate (1 mmol) and 2-aminopyrazine-3-carboxylic acid (2 mmol) and sodium hydroxide (2 mmol) were dissolved in a small volume of water to give a light yellow solution. Prismatic crystals separated from the solution after a few days.

### Refinement

Carbon-bound H-atoms were placed in calculated positions (C—H 0.93 Å) and were included in the refinement in the riding model approximation, with *U*(H) set to 1.2*U*(C).

The amino H-atoms and water H-atoms were located in a difference Fourier map, and were refined with a distance restraints of N–H 0.88±0.01 and O–H 0.84±0.01 Å; their temperature factors were tied to those of the parent atoms by a factor of 1.5 times.

The final difference Fourier map was featureless.

The second value in the WGHT is somewhat large. Using a smaller value led to a deeper hole in the final difference Fourier map and a larger *Goodness-of-fit*.

### Crystal data

**Table:**

[Mn(C_5_H_4_N_3_O_2_)_2_(H_2_O)_2_]·H_2_O	*F*(000) = 1576
*M**_r_* = 385.21	*D*_x_ = 1.681 Mg m^−^^3^
Orthorhombic, *F**d**d*2	Mo *K*α radiation, λ = 0.71073 Å
Hall symbol: F 2 -2d	Cell parameters from 4731 reflections
*a* = 8.3107 (6) Å	θ = 3.0–27.4°
*b* = 29.5862 (17) Å	µ = 0.92 mm^−^^1^
*c* = 12.3791 (7) Å	*T* = 293 K
*V* = 3043.8 (3) Å^3^	Prism, yellow
*Z* = 8	0.15 × 0.10 × 0.08 mm

### Data collection

**Table:**

Rigaku R-AXIS RAPID diffractometer	1684 independent reflections
Radiation source: fine-focus sealed tube	1086 reflections with *I* > 2σ(*I*)
graphite	*R*_int_ = 0.056
Detector resolution: 10.000 pixels mm^-1^	θ_max_ = 27.4°, θ_min_ = 3.0°
ω scans	*h* = −10→10
Absorption correction: multi-scan (*ABSCOR*; Higashi, 1995)	*k* = −38→38
*T*_min_ = 0.875, *T*_max_ = 0.930	*l* = −16→15
7239 measured reflections	

### Refinement

**Table:**

Refinement on *F*^2^	Hydrogen site location: inferred from neighbouring sites
Least-squares matrix: full	H atoms treated by a mixture of independent and constrained refinement
*R*[*F*^2^ > 2σ(*F*^2^)] = 0.047	*w* = 1/[σ^2^(*F*_o_^2^) + (0.0722*P*)^2^ + 15.3101*P*] where *P* = (*F*_o_^2^ + 2*F*_c_^2^)/3
*wR*(*F*^2^) = 0.165	(Δ/σ)_max_ = 0.001
*S* = 1.14	Δρ_max_ = 0.50 e Å^−^^3^
1684 reflections	Δρ_min_ = −0.90 e Å^−^^3^
126 parameters	Extinction correction: *SHELXL97* (Sheldrick, 2008), Fc^*^=kFc[1+0.001xFc^2^λ^3^/sin(2θ)]^-1/4^
6 restraints	Extinction coefficient: 0.0014 (3)
Primary atom site location: structure-invariant direct methods	Absolute structure: Flack (1983), 775 Friedel pairs
Secondary atom site location: difference Fourier map	Flack parameter: −0.02 (5)

### Fractional atomic coordinates and isotropic or equivalent isotropic displacement parameters (Å^2^)

**Table:**

	*x*	*y*	*z*	*U*_iso_*/*U*_eq_	
Mn1	0.2500	0.2500	0.53687 (11)	0.0378 (4)	
O1	0.0649 (6)	0.26569 (16)	0.4190 (4)	0.0457 (11)	
O2	−0.0500 (7)	0.31796 (16)	0.3169 (4)	0.0606 (16)	
O1W	0.0675 (8)	0.24512 (17)	0.6593 (4)	0.0561 (16)	
H11	0.084 (11)	0.225 (2)	0.706 (5)	0.084*	
H12	0.006 (9)	0.267 (2)	0.675 (8)	0.084*	
O2W	−0.2500	0.2500	0.5133 (9)	0.069 (3)	
H2	−0.164 (7)	0.248 (4)	0.478 (7)	0.104*	
N1	0.2472 (6)	0.32757 (14)	0.5175 (4)	0.0348 (13)	
N2	0.2176 (7)	0.41941 (18)	0.4774 (5)	0.0515 (16)	
N3	0.0251 (9)	0.4054 (2)	0.3489 (6)	0.0634 (19)	
H31	−0.048 (9)	0.391 (3)	0.311 (7)	0.095*	
H32	0.035 (11)	0.4344 (8)	0.335 (8)	0.095*	
C1	0.0468 (8)	0.30645 (19)	0.3886 (5)	0.0409 (14)	
C2	0.1439 (7)	0.34192 (19)	0.4439 (5)	0.0349 (12)	
C3	0.1271 (8)	0.3890 (2)	0.4229 (5)	0.0427 (14)	
C4	0.3184 (10)	0.4035 (2)	0.5501 (7)	0.0587 (19)	
H4	0.3808	0.4241	0.5886	0.070*	
C5	0.3368 (9)	0.3572 (2)	0.5727 (6)	0.0503 (17)	
H5	0.4095	0.3474	0.6248	0.060*	

### Atomic displacement parameters (Å^2^)

**Table:**

	*U*^11^	*U*^22^	*U*^33^	*U*^12^	*U*^13^	*U*^23^
Mn1	0.0457 (7)	0.0301 (6)	0.0376 (7)	0.0012 (7)	0.000	0.000
O1	0.051 (3)	0.035 (2)	0.051 (3)	−0.001 (2)	−0.009 (2)	0.000 (2)
O2	0.076 (4)	0.050 (2)	0.055 (4)	0.010 (2)	−0.035 (3)	0.000 (2)
O1W	0.070 (4)	0.048 (3)	0.050 (3)	0.010 (3)	0.019 (3)	0.008 (2)
O2W	0.044 (4)	0.055 (4)	0.109 (10)	−0.009 (4)	0.000	0.000
N1	0.041 (2)	0.030 (2)	0.033 (4)	−0.001 (2)	−0.009 (3)	0.003 (2)
N2	0.056 (4)	0.039 (3)	0.059 (4)	−0.015 (3)	−0.008 (3)	0.004 (3)
N3	0.075 (5)	0.042 (3)	0.073 (5)	−0.001 (3)	−0.029 (4)	0.019 (3)
C1	0.050 (4)	0.033 (3)	0.040 (3)	0.001 (3)	−0.001 (3)	−0.003 (3)
C2	0.041 (3)	0.034 (3)	0.030 (3)	0.002 (2)	−0.008 (3)	−0.003 (2)
C3	0.046 (4)	0.037 (3)	0.044 (4)	−0.001 (3)	−0.001 (3)	0.006 (3)
C4	0.063 (4)	0.045 (4)	0.068 (5)	−0.019 (3)	−0.014 (4)	0.013 (4)
C5	0.055 (4)	0.043 (3)	0.053 (4)	−0.007 (3)	−0.021 (3)	0.006 (3)

### Geometric parameters (Å, °)

**Table:**

Mn1—O1W^i^	2.149 (6)	N1—C5	1.338 (8)
Mn1—O1W	2.149 (6)	N2—C4	1.316 (10)
Mn1—O1	2.170 (5)	N2—C3	1.352 (8)
Mn1—O1^i^	2.170 (5)	N3—C3	1.339 (8)
Mn1—N1^i^	2.308 (4)	N3—H31	0.88 (7)
Mn1—N1	2.308 (4)	N3—H32	0.88 (3)
O1—C1	1.273 (7)	C1—C2	1.490 (8)
O2—C1	1.245 (8)	C2—C3	1.424 (8)
O1W—H11	0.84 (7)	C4—C5	1.409 (9)
O1W—H12	0.84 (7)	C4—H4	0.9300
O2W—H2	0.84 (7)	C5—H5	0.9300
N1—C2	1.322 (7)		
			
O1W^i^—Mn1—O1W	90.3 (4)	C5—N1—Mn1	126.3 (4)
O1W^i^—Mn1—O1	163.73 (16)	C4—N2—C3	117.2 (6)
O1W—Mn1—O1	89.33 (18)	C3—N3—H31	129 (7)
O1W^i^—Mn1—O1^i^	89.33 (18)	C3—N3—H32	115 (6)
O1W—Mn1—O1^i^	163.73 (16)	H31—N3—H32	115 (9)
O1—Mn1—O1^i^	95.5 (3)	O2—C1—O1	123.1 (6)
O1W^i^—Mn1—N1^i^	97.65 (18)	O2—C1—C2	119.0 (5)
O1W—Mn1—N1^i^	90.79 (18)	O1—C1—C2	117.9 (6)
O1—Mn1—N1^i^	98.61 (18)	N1—C2—C3	120.2 (5)
O1^i^—Mn1—N1^i^	73.16 (17)	N1—C2—C1	116.2 (5)
O1W^i^—Mn1—N1	90.79 (18)	C3—C2—C1	123.5 (5)
O1W—Mn1—N1	97.65 (18)	N3—C3—N2	116.9 (6)
O1—Mn1—N1	73.16 (17)	N3—C3—C2	122.7 (6)
O1^i^—Mn1—N1	98.61 (18)	N2—C3—C2	120.3 (6)
N1^i^—Mn1—N1	168.0 (3)	N2—C4—C5	123.5 (7)
C1—O1—Mn1	119.0 (4)	N2—C4—H4	118.2
Mn1—O1W—H11	115 (6)	C5—C4—H4	118.2
Mn1—O1W—H12	122 (7)	N1—C5—C4	118.4 (6)
H11—O1W—H12	119 (10)	N1—C5—H5	120.8
C2—N1—C5	120.2 (5)	C4—C5—H5	120.8
C2—N1—Mn1	113.4 (4)		
			
O1W^i^—Mn1—O1—C1	14.2 (12)	Mn1—N1—C2—C3	179.6 (5)
O1W—Mn1—O1—C1	102.9 (5)	C5—N1—C2—C1	−178.6 (6)
O1^i^—Mn1—O1—C1	−92.7 (5)	Mn1—N1—C2—C1	1.0 (7)
N1^i^—Mn1—O1—C1	−166.4 (5)	O2—C1—C2—N1	−178.5 (6)
N1—Mn1—O1—C1	4.7 (5)	O1—C1—C2—N1	3.0 (9)
O1W^i^—Mn1—N1—C2	179.9 (4)	O2—C1—C2—C3	3.0 (9)
O1W—Mn1—N1—C2	−89.8 (4)	O1—C1—C2—C3	−175.5 (6)
O1—Mn1—N1—C2	−2.8 (4)	C4—N2—C3—N3	−179.9 (8)
O1^i^—Mn1—N1—C2	90.4 (4)	C4—N2—C3—C2	−0.7 (10)
N1^i^—Mn1—N1—C2	44.8 (4)	N1—C2—C3—N3	179.6 (7)
O1W^i^—Mn1—N1—C5	−0.6 (6)	C1—C2—C3—N3	−1.9 (10)
O1W—Mn1—N1—C5	89.8 (6)	N1—C2—C3—N2	0.5 (10)
O1—Mn1—N1—C5	176.8 (6)	C1—C2—C3—N2	178.9 (6)
O1^i^—Mn1—N1—C5	−90.0 (6)	C3—N2—C4—C5	0.5 (13)
N1^i^—Mn1—N1—C5	−135.6 (5)	C2—N1—C5—C4	−0.2 (10)
Mn1—O1—C1—O2	175.7 (5)	Mn1—N1—C5—C4	−179.8 (5)
Mn1—O1—C1—C2	−5.8 (8)	N2—C4—C5—N1	0.0 (13)
C5—N1—C2—C3	0.0 (9)		

Symmetry codes: (i) −*x*+1/2, −*y*+1/2, *z*.

### Hydrogen-bond geometry (Å, °)

**Table:**

*D*—H···*A*	*D*—H	H···*A*	*D*···*A*	*D*—H···*A*
O1w—H11···O2^ii^	0.84 (7)	1.89 (3)	2.704 (7)	162 (9)
O1w—H12···N2^iii^	0.84 (7)	2.02 (4)	2.792 (7)	152 (9)
O2w—H2···O1	0.84 (7)	2.10 (4)	2.902 (7)	159 (10)
N3—H31···O2	0.88 (7)	2.17 (9)	2.690 (8)	118 (8)
N3—H32···O2w^iv^	0.88 (3)	2.15 (3)	3.001 (7)	161 (9)

Symmetry codes: (ii) −*x*, −*y*+1/2, *z*+1/2; (iii) *x*−1/4, −*y*+3/4, *z*+1/4; (iv) −*x*−1/4, *y*+1/4, *z*−1/4.
